# Chemoradiotherapy response in recurrent rectal cancer

**DOI:** 10.1002/cam4.169

**Published:** 2013-12-16

**Authors:** Stanley K T Yu, Aneel Bhangu, Diana M Tait, Paris Tekkis, Andrew Wotherspoon, Gina Brown

**Affiliations:** 1 Radiotherapy Department, Royal Marsden NHS Foundation TrustSutton, London, UK; 2 Academic Surgery Department, Royal Marsden NHS Foundation TrustFulham Road, London, UK; 3 Histopathology Department, Royal Marsden NHS Foundation TrustLondon, UK; 4 Radiology Department, Royal Marsden NHS Foundation TrustLondon, UK

**Keywords:** Chemoradiotherapy, Kaplan–Meier estimate, magnetic resonance imaging, neoplasm recurrence, rectal neoplasms, treatment outcome

## Abstract

The efficacy of response to preoperative chemoradiotherapy (CRT) in recurrent versus primary rectal cancer has not been investigated. We compared radiological downsizing between primary and recurrent rectal cancers following CRT and determined the optimal size reduction threshold for response validated by survival outcomes. The proportional change in tumor length for primary and recurrent rectal cancers following CRT was compared using the independent sample *t*-test. Overall survival (OS) was calculated using the Kaplan–Meier product limit method and differences between survival for tumor size reduction thresholds of 30% (response evaluation criteria in solid tumors [RECIST]), 40%, and 50% after CRT in primary and recurrent rectal cancer groups. A total of 385 patients undergoing CRT were analyzed, 99 with recurrent rectal cancer and 286 with primary rectal cancer. The mean proportional reduction in maximum craniocaudal length was significantly higher for primary rectal tumors (33%) compared with recurrent rectal cancer (11%) (*P* < 0.01). There was no difference in OS for either primary or recurrent rectal cancer when ≤30% or ≤40% definitions were used. However, for both primary and recurrent tumors, significant differences in median 3-year OS were observed when a RECIST cut-off of 50% was used. OS was 99% versus 77% in primary and 100% versus 42% in recurrent rectal cancer (*P* = 0.002 and *P* = 0.03, respectively). Only patients that demonstrated >50% size reduction showed a survival benefit. Recurrent rectal cancer appears radioresistant compared with primary tumors for tumor size after CRT. Further investigation into improving/intensifying chemotherapy and radiotherapy for locally recurrent rectal cancer is justified.

## Introduction

Improvements in staging and surgical technique have drastically reduced pelvic recurrence rates in rectal cancer. However, 3–5% of patients still develop local recurrence after primary surgery 1,2 with devastating consequences such as sacral and perineal pain or obstruction. The median survival of untreated patients with recurrent rectal cancer is 3–8 months 3.

The use of preoperative chemoradiotherapy (CRT) and radiotherapy treatment regimens in recurrent rectal cancer have been extrapolated from studies in primary rectal cancer showing improvements in resectability, local control, and survival rate 4–7. However, currently, there are neither good data from prospective randomized studies regarding optimum preoperative treatments for recurrent rectal cancer nor is there data regarding assessing the efficacy of response to any such treatments. Furthermore, there are no comparisons of primary versus recurrent rectal cancer response rates to CRT and therefore whether extrapolation of dose and regimes are appropriate. Recurrent rectal cancer cannot be easily classified using TNM (Union for International Cancer Control Classification: Tumor, Node, Metastasis) and studies to date have provided staging assessments largely based on anatomic extent 8 and surgical resectability prior to surgical exenteration, which cannot easily be measured to judge response.

Measurement of tumor size is arguably a logical starting point to assess tumor response, but published criteria to date are subject to controversy and lack outcomes validation. In 1977, World Health Organization (WHO) proposed a 50% reduction in either bidimensional or unidimensional measurements as a pragmatic assessment of tumor response. In later years this was superseded by response evaluation criteria in solid tumors (RECIST) 9 1.1 indicating that a >30% reduction in size should be defined as a partial response. Very few validations exist for RECIST criteria against outcomes in rectal cancer and none for recurrent rectal cancer. A recent study evaluating tumor response in Phase I trials demonstrated that tumor response could be considered as a continuous variable that correlates linearly with outcomes 10,11. It could be seen from this data that the standard RECIST 30% cut-off for response has a tendency to overestimate responders and that the best outcomes are seen when response is 45% or more 12,13. If a response threshold can be validated for rectal cancers, then this may better assist in the objective assessment of response in patients undergoing treatment for pelvic recurrent disease.

This study attempts to define a clinically meaningful tumor shrinkage threshold and its corresponding survival benefit in patients with primary and recurrent rectal cancer and to compare any differences in CRT response rates for these two groups of patients.

These results may enable future studies to evaluate CRT regimens and radiotherapy doses in patients with recurrent rectal cancer.

## Methods

The project was a cohort analysis of all patients treated with preoperative CRT at a single institution for either locally advanced or recurrent rectal cancer between 2003 and 2011. The study protocol was approved as a service evaluation study by the Local Institutional Ethical and Research Review Board. As the project was noninterventional, patient consent was not required.

The primary aim of this study was to determine the difference in radiological downsizing response between primary locally advanced rectal cancer and local recurrent rectal cancer (CRT naïve) following CRT and, second, to determine the optimal RECIST threshold for response validated by survival outcomes. Based on independent sample *t* testing, a sample size of at least 50 patients in each group would be required to detect a 19% (72% 14 vs. 53%) difference in mean reduction in tumor size following CRT with 80% power.

### Preoperative clinical assessment and treatments

Patients diagnosed with primary rectal cancer and recurrent rectal cancer underwent clinical examination, colonoscopy, high-resolution MRI pelvis 15, and CT chest, abdomen, and pelvis (CT CAP) as staging. The clinical, radiological, and histological results were reviewed in the multidisciplinary team (MDT) meeting.

For primary tumors, rectal cancer stage was confirmed.

For recurrences, local extent and compartmental involvement were recorded and a treatment plan was recommended.

All patients included in this study received CRT using 50.4–54.0 Gy/28–30 fractions with Capecitabine 825 mg/m^2^ twice daily during RT.

Reassessment high-resolution MRI and CT CAP scans were acquired at a minimum of 4 weeks following completion of CRT and restaged at the MDT meeting prior to further management. Maximum two-dimensional craniocaudal lengths of the rectal tumors and recurrent tumors were measured before and after CRT using high-resolution MRI.

### Histopathology

All specimens were evaluated according to the Royal College of Pathologists' protocol 16. The specimens were received fresh, and fixed in 10% formalin. After the specimen is fixed, the segments of bowel including the tumor, the 30 mm segment of intestine proximally and distally to the tumor, and the attached mesentery were sectioned transversely at 3–4 mm intervals with a sharp knife to produce slices that included the tumor, the adjacent lymph nodes, and the serosal and nonperitonealized resection margins 16. Slices were embedded in blocks and processed for hematoxylin and eosin staining in 5 mm sections. If no definite residual tumor could be recognized, the whole of the tumor site/scar was blocked for histology. The ypT category was recorded. Pathological complete response (pCR) was defined as the absence of any tumor cells in pathology specimen defined as ypT0N0.

### Statistical analysis

The proportional change in tumor size in primary rectal cancer and local recurrent rectal cancer was compared using the independent sample *t*-test.

The proportional change in tumor length before and after CRT by MRI was compared for the primary rectal and recurrent rectal cancer groups. The tumor size reduction was calculated as (baseline—posttreatment craniocaudal lengths)/baseline craniocaudal length.

Overall survival (OS) was calculated using the Kaplan–Meier product limit method and differences between survival for tumor size reduction thresholds of 30% (RECIST) 9, 40%, and 50% after CRT in primary rectal cancer and recurrent rectal cancer groups. The differences in survival were tested for significance using the Mantel–Cox log-rank test.

For patients in the primary rectal cancer and recurrent rectal cancer group, OS was measured from the date of diagnosis of primary cancer or recurrent rectal cancer, respectively, to the date of event, that is, death.

For all analyses, a *P*-value less than 5% was considered significant.

### Inclusion criteria

Patients with biopsy proven primary rectal cancer who received neoadjuvant CRT and patients with recurrent rectal cancer who were CRT naive were included in the study.

Inclusion criteria were as follows: biopsy-proven primary rectal cancer patients who had received neoadjuvant CRT, recurrent rectal cancer patients receiving CRT, WHO performance status 0–2.

Patients with recurrent rectal cancer were CRT naïve due to stage and clinical reasons at initial presentation. The clinical reasons why patients had not undergone CRT and later presented with recurrent rectal cancer were as follows:

1. Patient choice.
2. Requiring immediate surgery due to emergency.
3. In 2003–2006, MRI was not used to determine the optimal plane of surgery for abdominoperineal excision; therefore, there were high rates of positive margins due to less than optimal surgical planning and the use of CRT, this has subsequently been addressed in low rectal cancer trial 17–19.

### Exclusion criteria

Patients less than 18 years old.

Received short-course preoperative radiotherapy (SCPRT) or RT alone.

Did not undergo MRI before and after CRT.

Had incomplete histology data.

Received adjuvant CRT and other malignancy.

## Results

In total, 385 patients with primary or recurrent rectal cancer who underwent CRT between 2003 and 2011 were included in the study, 99 of whom had recurrent rectal cancer and the remaining 286 had primary locally advanced rectal cancer. A total of 50/286 patients with primary rectal cancer (17% with 95% CI 11–19%) developed pCR after CRT. Forty-three patients with recurrent rectal cancer were excluded from analysis: As a repeat MRI following CRT was not performed, all these patients were treated with palliative intent.

Patient demographics and characteristics are shown in Table 1. The median follow-up period for the cohort was 43 months. There was no significant difference in baseline characteristics of patients with primary rectal cancer and those of the original primary rectal cancer prior to primary surgery/recurrence for recurrent rectal cancer patients.

**Table 1:** Patients' demographics with primary rectal cancer and recurrent rectal cancer treated with CRT between 2003 and 2009.

Variable	Primary rectal cancer	Recurrent rectal cancer	*P*-value
Gender	Total = 286	Total = 56	
Male	176	38	
Female	110	18	0.32
Age			
>50	43	11	
>50	243	45	0.37
Median time to MRI	29 days (25th and 75th percentile: 28–33 days)	30 days (25th and 75th percentile: 27–35 days)	
Pretreatment tumor size (MRI staging) ≤4 cm	120 (42%)	32 (57%)	
>4 cm	166 (58%)	24 (43%)	**0.04**
Primary staging T-stage (MR) or original primary tumor stage (for recurrence)			
≤mrT2	119 (42%)	23 (41%)	
>mrT2	167 (58%)	33 (59%)	1.00
mrN0	201 (70%)	36 (64%)	
mrN1/N2	85 (30%)	20 (36%)	0.43
Primary tumor distance from anal verge (by MRI)			
>5 cm	194 (68%)	40 (71%)	
≤5 cm	92 (32%)	16 (29%)	0.64
Primary tumor EMVI status by MRI			
Negative	247 (86%)	47 (84%)	
Positive	39 (14%)	9 (16%)	0.67
Primary tumor CRM status by MRI			
Negative	269 (94%)	54 (96%)	
Positive	17 (6%)	2 (4%)	0.75
MRI response after CRT (size reduction)			
0–30%	137	34	
31–49%	79	14	
>50%	70	8	0.15
Median 3 years OS in			
>30%	82%	49%	
≤30%	83% (*P* = 0.57)	36% (*P* = 0.28)	
>40%	83%	53%	
≤40%	81% (*P* = 0.28)	39% (*P* = 0.36)	
>50%	**96% (95% CI = 90–102%)**	**100% (95% CI = 84–116%)**	
≤50%	**77% (95% CI = 71–83%) (*****P***** = 0.002)**	**42% (95% CI = 27–57%) (*****P***** = 0.03)**	
Site of tumor recurrent			
Anastomotic site		18 (32%)	
Nonanastomotic site		38 (68%)	

CRM, circumferential resection margin; CRT, chemoradiotherapy; EMVI, extramural vascular invasion; OS, overall survival.Bold values indicate statistically significant.

The median time for MRI after CRT was 29 days (25th and 75th percentile: 28–33 days) in patients with primary rectal cancer and 30 days (25th and 75th percentile: 27–35 days) in patients with recurrent rectal cancer.

Of the 56 recurrent rectal cancer patients who underwent CRT, 30 (54%) underwent surgery. Eleven of 30 (37%) patients who underwent surgery had R1 resections and 19/30 patients (63%) had R0 complete resections. Five of 56 (9% with 95% CI 2–17%) patients with recurrent rectal cancer developed pCR following CRT.

The mean rectal tumor size was significantly larger (0.58 cm vs. 0.43 cm with *P* = 0.04) for primary tumor compared with recurrent tumors. The mean proportional reduction in maximum craniocaudal length was significantly higher for primary rectal tumors (33%) when compared with recurrent rectal cancer (11%) after CRT (*P* < 0.01). Hence, recurrent rectal cancer was significantly less responsive in terms of tumor size reduction after CRT when compared with primary rectal cancers.

### Survival analysis

**Figure 1:**
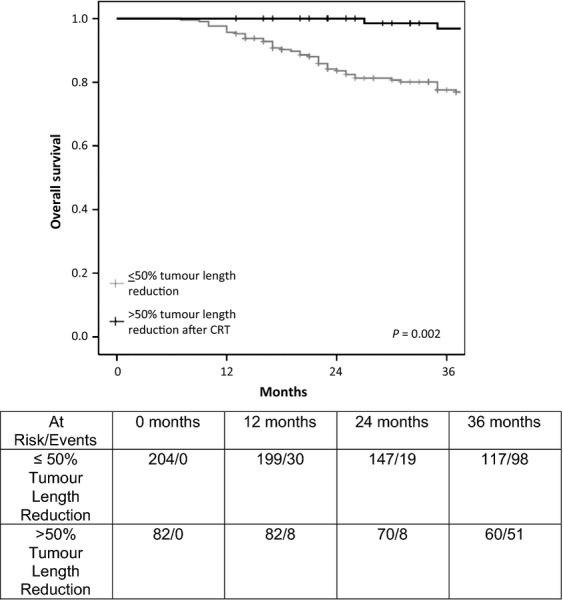
Overall survival in patients with primary rectal cancer who underwent chemoradiotherapy (CRT).

**Figure 2:**
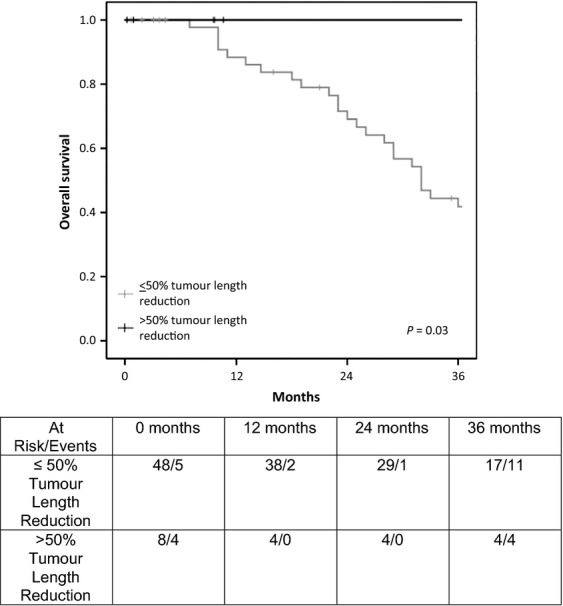
Overall survival in patients with recurrent rectal cancer who underwent chemoradiotherapy (CRT).

There was no difference in OS for either in primary or recurrent rectal cancer when either *a* ≤ 30% or ≤40% RECIST definition was used for tumor size reduction after CRT. However, for both primary and recurrent tumors, significant differences in median 3-year OS were observed when a RECIST cut-off of 50% was used (*P* = 0.002 and *P* = 0.03, respectively) (Figs. 1 and 2). Patients with primary rectal cancer who underwent CRT were 2.4 times more likely (*P* = 0.03) to develop >50% craniocaudal length reduction following CRT when compared with patients with recurrent rectal cancer.

## Discussion

Our study has shown that recurrent rectal cancers appear to be relatively radioresistant compared with primary rectal cancer and this has not been previously demonstrated. There was a difference in the rates of pCR between the primary and recurrent tumor. Although this has not reached statistical significance, it is interesting to know that the pCR rate for recurrent cancer was only 9% (with 95% CI 2–17%), which is substantially lower than the known published rates for pCR in primary rectal cancer. This adds more evidence to our overall findings that recurrent rectal cancers are relatively chemoradioresistant. It may therefore be reasonable to question current practice based on an assumption that response rates to CRT in recurrent and primary rectal cancer should be equivalent. The commonest CRT regimen used for both recurrent and primary rectal cancer is 45–50.4 Gy in 25–28 fractions with fluorouracil (5FU)-based chemotherapy 20.

Rodel et al. 21 has showed, in a retrospective study of 35 patients with recurrent rectal cancer, that using “standard” CRT (50.4 Gy) and 5FU chemotherapy followed by extensive curative surgery with R0 resection can improve the 3 years survival from 39% to 82% when compared with patients who did not undergo surgery. Eighty per cent of the recurrent rectal cancer patients became resectable. Seventeen patients (61%) achieved R0 resection in surgery and patients with R0 resection carried significant survival benefit. There are few phase I and II studies investigating into means of intensifying CRT regimen in recurrent rectal cancer. Hu et al. 22 showed that using two cycles of FOLFOX 4 concurrent chemotherapy (and two to four cycles of FOLFOX 4 after CRT) with radiotherapy dose of up to 60 Gy in 48 patients with recurrent tumor carried better survival than RT alone and the regimen is well tolerated. Caravatta et al. 23 showed that using high-dose radiotherapy (55 Gy in 25 fractions) with oxaliplatin and raltitrexed could be safely administrated to patients with recurrent rectal cancer. Intraoperative brachytherapy (IORT) using 10–20 Gy in total, which has been adopted by some centers for treatment in recurrent rectal cancer, remains controversial with limited data regarding benefit and outcomes 24. For example, Guo et al. 25 showed that IORT (with a median dose of 14.4 Gy) was effective in treating recurrent rectal cancer. Three years median survival was 43%. Local failure rate after IORT and surgery was 32%. In contrast, Turley et al. 26 investigated 29 patients using IORT with a median dose of 12–15 Gy and showed that 11 patients (38%) developed long-term morbidity including long-term wound complication, ureter obstruction, and fistulae. To sum up, CRT with two concurrent chemotherapy is well tolerated and has better survival rate when compared with RT alone in recurrent rectal cancer and higher RT dose appears tolerable. Our results have shown that recurrent rectal cancer is relatively resistant to conventional doses of CRT; therefore, further investigation into more intensive preoperative treatment for recurrent rectal cancer is warranted if better radiological response rates and overall outcomes are to be achieved.

Most staging systems for recurrent rectal cancer to date have not been shown to be predictive for local control or OS and there has been no previous agreed definition of response assessment in recurrent rectal cancer 27. However, recent preliminary data suggest that the number of compartments and the anatomical location of recurrence are of prognostic relevance 8. Our study has now shown that the conventional RECIST definition for response of 30% substantially overestimates the efficacy of response in terms of overall outcomes. On the other hand, for both primary and recurrent rectal cancers, a 50% reduction in size after treatment gives the best prediction for good survival and is thus the best measure for the effectiveness of preoperative treatment. This illustrates the importance of validating response criteria against survival outcomes and we propose that a craniocaudal length reduction of >50% after CRT should be considered as an appropriate definition of “true partial response” for both primary and recurrent rectal cancer.

A limitation of this study was that response assessment following CRT using MRI was not measured using mrTumour Regression Grade (mrTRG). More recently, an mrTRG system has been developed which predicts survival outcomes and shows good correlation with histopathology response following preoperative therapy in primary rectal cancer after CRT 13,28. It is possible that a mrTRG system would also be of prognostic relevance in recurrent rectal cancer, but this needs to be tested along with our proposed definition of 50% rather than the conventional 30% “RECIST” in future studies.

### Summary

Locally recurrent rectal cancers have devastating consequences including sacral/perineal pain and obstruction. Patients with primary and recurrent rectal cancers who demonstrate *a* >50% reduction in craniocaudal length following CRT have a survival benefit. Recurrent rectal cancer is 2.4 times less likely to show *a* >50% reduction in craniocaudal tumor length following CRT. Hence, recurrent rectal cancers appear to be relatively radioresistant compared with primary rectal cancers. Further investigations into improving clinical relevant staging system, response assessment, and improving/intensifying chemotherapy and radiotherapy response in local recurrent rectal cancer are much needed.

## Conflict of Interest

None declared.
